# Aldehyde dehydrogenase activity plays a Key role in the aggressive phenotype of neuroblastoma

**DOI:** 10.1186/s12885-016-2820-1

**Published:** 2016-10-10

**Authors:** Marjorie Flahaut, Nicolas Jauquier, Nadja Chevalier, Katya Nardou, Katia Balmas Bourloud, Jean-Marc Joseph, David Barras, Christian Widmann, Nicole Gross, Raffaele Renella, Annick Mühlethaler-Mottet

**Affiliations:** 1 Pediatric Hematology-Oncology Research Laboratory, Pediatric Division, University Hospital CHUV, Lausanne, Switzerland; 2 Pediatric Surgery, Pediatric Division, University Hospital CHUV, Lausanne, Switzerland; 3 Department of Physiology, University of Lausanne, Lausanne, Switzerland; 4 SIB Swiss Institute of Bioinformatics, Bioinformatics Core Facility, Lausanne, Switzerland

## Abstract

**Background:**

The successful targeting of neuroblastoma (NB) by associating tumor-initiating cells (TICs) is a major challenge in the development of new therapeutic strategies. The subfamily of aldehyde dehydrogenases 1 (ALDH1) isoenzymes, which comprises ALDH1A1, ALDH1A2, and ALDH1A3, is involved in the synthesis of retinoic acid, and has been identified as functional stem cell markers in diverse cancers. By combining serial neurosphere passages with gene expression profiling, we have previously identified ALDH1A2 and ALDH1A3 as potential NB TICs markers in patient-derived xenograft tumors. In this study, we explored the involvement of ALDH1 isoenzymes and the related ALDH activity in NB aggressive properties.

**Methods:**

ALDH activity and ALDH1A1/A2/A3 expression levels were measured using the ALDEFLUOR™ kit, and by real-time PCR, respectively. ALDH activity was inhibited using the specific ALDH inhibitor diethylaminobenzaldehyde (DEAB), and ALDH1A3 gene knock-out was generated through the CRISPR/Cas9 technology.

**Results:**

We first confirmed the enrichment of ALDH1A2 and ALDH1A3 mRNA expression in NB cell lines and patient-derived xenograft tumors during neurosphere passages. We found that high ALDH1A1 expression was associated with less aggressive NB tumors and cell lines, and correlated with favorable prognostic factors. In contrast, we observed that ALDH1A3 was more widely expressed in NB cell lines and was associated with poor survival and high-risk prognostic factors. We also identified an important ALDH activity in various NB cell lines and patient-derived xenograft tumors. Specific inhibition of ALDH activity with diethylaminobenzaldehyde (DEAB) resulted in a strong reduction of NB cell clonogenicity, and TIC self-renewal potential, and partially enhanced NB cells sensitivity to 4-hydroxycyclophosphamide. Finally, the specific knock-out of *ALDH1A3* via CRISPR/Cas9 gene editing reduced NB cell clonogenicity, and mediated a cell type-dependent inhibition of TIC self-renewal properties.

**Conclusions:**

Together our data uncover the participation of ALDH enzymatic activity in the aggressive properties and 4-hydroxycyclophosphamide resistance of NB, and show that the specific ALDH1A3 isoenzyme increases the aggressive capacities of a subset of NB cells.

**Electronic supplementary material:**

The online version of this article (doi:10.1186/s12885-016-2820-1) contains supplementary material, which is available to authorized users.

## Background

Neuroblastoma (NB), which arises from neural crest-derived sympatho-adrenal progenitors, is one of the most life-threatening solid tumors of childhood [1–3]. The hallmark of NB is its extreme biological, genetic, and clinical heterogeneity. This leads to a broad spectrum of clinical outcomes, ranging from spontaneous regression to an aggressive life-threatening disease for high-risk NB, with only 40 % long-term survival despite intensive multimodal therapy [1–3]. While only few recurrent gene mutations have been found in NB tumors, a large number of recurrent somatic genetic alterations have been described, which includes numerical or segmental chromosomal alterations [1, 2, 4–6].

Like their tumor of origin, NB cell lines display important biological heterogeneity. Three cell subtypes arise spontaneously in NB cell line cultures: a) neuroblastic (N-type), displaying properties of embryonic sympathoblasts, b) substrate-adherent (S-type), resembling Schwannian, glial or melanocytic progenitor cells, and c) intermediate (I-type) subtype [7]. I-type cells express markers of both N and S subtypes and display bidirectional differentiation potential when treated with specific agents [8–10]. Moreover, I-type cells are significantly more aggressive than N- or S-type cells, and were proposed to represent NB stem cells (SCs) or malignant neural crest SCs [9, 11].

In recent years, emerging evidence has suggested that tumor progression, metastasis, and chemotherapeutic drug resistance are driven by a minor cell subpopulation, designed as cancer stem cells (CSCs) or tumor-initiating cells (TICs) [12–14]. These are capable of self-renewal and differentiation into heterogeneous phenotypic and functional lineages, and are characterized by plasticity [14–16]. In a previous study aiming to identify NB TIC markers, we combined serial neurosphere (NS) passage assays, which allow the enrichment of TICs, with gene expression profiling. This allowed the identification of a gene expression signature associated to NB TICs [17]. Among this gene profile, ALDH1A2 and ALDH1A3 were selected for further investigations of their role in maintaining NB TIC properties. The rationale behind this selection is based on the demonstration of the implication of ALDH activity in the biology of normal SCs and CSCs in other settings [18–21].

ALDHs belong to a superfamily of 19 genes coding for NAD(P)^+^-dependent enzymes involved in the detoxification of a large number of endogenous and exogenous aldehydes [22, 23]. The ALDH1 subfamily, which includes A1, A2 and A3 isoforms, is involved in the synthesis of retinoic acid, playing therefore an important role in developing tissues [22]. Elevated ALDH activity was first demonstrated in normal hematopoietic progenitor/stem cells and is now commonly used for the isolation of CSCs in multiple tumor settings [24, 25]. Moreover, several ALDH isoenzymes were associated to TICs properties, such as ALDH1A1 in melanoma and lung adenocarcinoma [20, 26], ALDH1B1 in colon cancer [27], ALDH1A3 in breast cancer and NSCLC [28, 29], and ALDH7A1 in prostate cancer [30]. ALDH1 expression was also correlates with cyclophosphamide resistance [23, 31, 32], a chemotherapeutic drug widely used for the treatment of many cancers, including NB. So far, ALDH activity has not been linked to NB tumor initiation or progression. However, a recent paper described the involvement of ALDH1A2 in the regulation of CSC properties in NB [33].

In this study, we aimed at exploring the expression pattern of the three ALDH1 isoforms in NB cell lines and patient-derived xenograft (PDX) tumors. ALDH activity was found to play a role in NB cell aggressive properties, such as clonogenicity, TIC proliferation, and cyclophosphamide resistance. In addition, we revealed that ALDH1A3 is associated with poor prognosis, and *ALDH1A3* gene disruption negatively impacted the aggressiveness of a subset of NB cell lines, suggesting that it can enhance NB tumorigenic properties.

## Methods

### Ethics statement

All in vivo procedures were performed under the guidelines of the Swiss Animal Protection Ordinance and the Animal Experimentation Ordinance of the Swiss Federal Veterinary Office (FVO). Animal experimentation protocols were approved by the Swiss FVO (authorization number: 1564.6). All reasonable efforts were made to ameliorate suffering, including anesthesia for painful procedures.

### Patient-derived xenograft

Tumor material was collected from NB patients, diagnosed in the Hemato-Oncology Unit of the University Hospital of Lausanne (Switzerland), after informed consent and in agreement with local institutional ethical regulations (Protocol 26/05, 07/02/2005). Patient-derived xenografts (PDX) NB1, NB2 and NB4 were produced by in vivo serial subcutaneous transplantations of bone-marrow derived tumor cells in athymic Swiss nude mice (Crl:NU(Ico)-Foxn1nu) from Charles River Laboratory (France) [17]. PDX tumors were dissociated as previously described [17].

### Cell culture

All well-characterized NB cell lines [34–36] were grown in Dulbecco’s modified Eagle’s medium (DMEM) (Gibco, Paisley, UK) supplemented with 10 % Fetal Bovine Serum (FBS) (Sigma-Aldrich, St Louis, USA) and 1 % penicillin/streptomycin (Gibco). The NB1-C cell line [17] was established from the dissociated NB1 PDX tumor derived from bone marrow metastatic cells (stage 4, NMYC not amplified). NB1-C cells were maintained in Neural Basic Medium (NBM) [DMEM/F12 supplemented with 1 % penicillin/streptomycin, 2 % B27 (Invitrogen, Carlsbad, USA), 20 ng/ml human recombinant bFGF (Peprotech, Rocky Hill, USA), and 20 ng/ml EGF (Peprotech)].

### RNA extraction, reverse transcription, and PCR

Total RNAs from NB cells (1 × 10^6^ cells) was obtained using the RNeasy Mini kit (Qiagen, Hilden, Germany) according to the manufacturer’s instructions. RNAs (0.2-1 μg) were reverse transcribed with the PrimeScript^TM^ RT reagent Kit (TAKARA Bio, St.Germain-en-Laye, France) using random primers and oligo dT primers according to the manufacturer’s instructions.

The expression levels of ALDH1A1, ALDH1A2, ALDH1A3, and MYC mRNAs were measured by real-time PCR using specific primers (QuantiTect primer assay, Qiagen), QuantiFast SYBRgreen assay (Qiagen), and the Corbett Rotor-Gene 6000 real-time cycler (Qiagen), as previously described [37]. The cycling conditions comprised 3 min polymerase activation at 95 °C, followed by 40 cycles of 3 s at 95 °C, 20s at 60 °C and 1 s at 72 °C for fluorescence acquisition. The ratio of each gene of interest to *HPRT1* gene expression and the relative gene expression were evaluated using the ΔCt and ΔΔCt methods, respectively.

NANOG, SOX2, and MYC expression levels were analyzed by PCR using GoTaq Hot Start Kit (Promega, Madison, USA) with the following primers: NANOG-for 5’-CAGCCCCGATTCTTCCACCAGTCCC-3’, NANOG-rev 5’-CGGAAGATTCCCAGTCGGGTTCACC-3’, SOX2-for 5’GGGAAATGGGAGGGGTGCAAAAGAGG-3, SOX2-rev 5’-TTGCGTGAGTGTGGATGGGATTGGTG-3’, MYC-for 5’-GCGTCCTGGGAAGGGAGATCCGGAGC-3’, MYC-rev 5’-TTGAGGGGCATCGTCGCGGGAGGCTG-3’. Cycling reactions were 2 min at 95 °C followed by 35 cycles of 30s at 95 °C, 30s at 60 °C and 30s at 72 °C, and 5 min at 72 °C.

### ALDEFLUOR assay

ALDH activity was analyzed using the ALDEFLUOR™ kit according to manufacturer’s instructions (Stem Cell Technologies, Grenoble, France). Briefly, 1×10^6^ NB cells or PDX-dissociated cells were resuspended in 1 ml ALDEFLUOR assay buffer. The ALDH substrate BODIPY™-aminoacetaldehyde (BAAA) was added to the cells. Immediately after mixing, half of the suspension was used as the negative control, by adding 5 μl of the ALDH inhibitor diethylaminobenzaldehyde (DEAB, 3 μM). Cells were incubated for 30–45 minutes at 37 °C, then washed twice, and suspended in ALDEFLUOR™ assay buffer containing 1 μg/ml of DAPI (Life Technologies, Switzerland) for viable cells selection. The brightly fluorescent ALDH^+^ cells were detected in the FL1-channel of a Gallios^TM^ Flow Cytometer (Beckman Coulter, Inc., USA) and data were analyzed using KALUZA™ software (Beckman Coulter, Inc., USA).

### ALDH activity inhibition

Cells were pre-treated with DEAB (Sigma-Aldrich) at 50 or 100 μM according to the cell line tested, or for control cells with Dimethyl sulfoxide (DMSO, Sigma-Aldrich) for 3 days prior functional assays, while maintaining the same amount of DEAB or DMSO during the assay.

### Proliferation assay

Cell proliferation was assessed using the MTS/PMS cell proliferation kit (Promega). Briefly, 10^4^ SK-N-Be2c cells and 2*10^4^ NB1-C cells per well were seeded in a 96-wells plate in DMEM/FCS or in NBM, respectively. Proliferation was monitored by measuring the OD at 405 nm immediately after seeding and after 24, 48, 72 and 96 h in presence of DEAB or DMSO for ALDH inhibition experiments, or without treatment.

### Methylcellulose clonogenic assay

Clonogenic assays in semi-solid conditions were performed as described [38]. Briefly, NB cells (1*10^3^) were grown in 500 μl semi-solid medium containing 53 % methylcellulose (Fluka), and 47 % DMEM/FCS (for SK-N-Be2c) or 47 % NBM (for NB1-C) in poly-Hema (poly2-hydroxyethyl methylacrylate, 16 mg/ml in EtOH; Sigma-Aldrich) -coated 24-wells plates. For ALDH activity inhibition assay, DEAB or DMSO was added in the semi-solid medium, and supplemented every 4 days in 100 μl of medium. After 2 weeks, colonies were counted using an optic microscope (Olympus, Volketswil, Switzerland).

### NB neurosphere culture and self-renewal assay

NS culture was performed as described in neural crest stem cell medium (NCSCm) in poly-Hema-coated six wells plates to prevent cell adhesion [17, 39]. For the production of serial NS passages, NB cells (1×10^5^ cells/ml) were plated, and spheres were dissociated every 7 days in 0.05 % trypsin-EDTA (Invitrogen), subsequently inhibited with Trypsin inhibitor (v/v) (Sigma-Aldrich). At each sphere passage, a part of the dissociated cells were tested for ALDH activity and for ALDH1A1/2/3 mRNA expression. For self-renewal assay, 1×10^4^ cells were plated in 500 μl NCSCm in triplicates without treatment, or in presence of DEAB or DMSO for ALDH inhibition experiments.

### Cell viability assays

Cells (2×10^4^ for SK-N-Be2c, IGR-N91 and IGR-N91R, or 4*10^4^ for NB1-C) were plated in 96-wells plates 24 h before treatment with 4-hydroxycyclophosphamide (4-HCPA, Niomech, Bielefeld, Germany) for 48 h. Cell viability was measured in quadruplicates using the MTS/PMS cell proliferation kit from Promega according to manufacturer’s instructions as described [40].

### ALDH1A3 knock out through CRISPR/Cas9 technology

Two sgRNAs targeting the early exon of the *ALDH1A3* gene were chosen in the published sgRNA library [41]. Oligos were designed as follow: sgALDH1A3.1: forward 5’-CACCGCTACATGTAACCCTTCAACT-3’, reverse 5’-AAACAGTTGAAGGGTTACATGTAGC-3’, sgALDH1A3.2: forward 5’- CACCGCGCTCAGCCCGACGTGGACA-3’, reverse 5’- AAACTGTCCACGTCGGGCTGAGCGC-3’. The lentiviral vector lentiCRISPR v2 [42] was obtained from Adgene (Cambridge, USA). LentiCRISPR v2-sgALDH1A3 plasmids were constructed according to the manufacturer’s instructions (Adgene). Virus production and lentiviral infections were performed as previously described [43] with the following modification: pCMVDR8.91 was replaced by psPAX2 (Adgene). Transduced SK-N-Be2c and NB1-C cells were selected 24 h post-infection with 5 μg/ml or 1 μg/ml of puromycin (Sigma-Aldrich), respectively. Control cells were transduced with virus containing the empty lentiCRIPR v2 vector. Clone isolation was performed by limiting dilution in 96-wells plate.

Validation of the ALDH1A3 KO by immunoblotting could not be performed due to the detection (using 3 different anti-ALDH1A3 antibodies) of a non-specific band migrating with a similar velocity as ALDH1A3 in the negative control SH-EP cell line lacking ALDH1A3 mRNA expression. Thus, genome editing in clones was verified by NGS sequencing. PCR amplicons were designed across the *ALDH1A3* genomic regions targeted by the sgRNAs to examine generation of indels. A second PCR was performed to attach Illumina adaptors and barcodes to samples according to manufacturer’s instructions. Primers for the second PCR include both a variable length sequence to increase library complexity and an 8 bp barcode for multiplexing of different biological samples. Amplicons were gel extracted, quantified, mixed and sequenced with a MiSeq SR300 (Illumina Inc., San Diego, USA). Sequencing reads were then processed with the following bioinformatic tools to quantify the occurrence of indels in the selected clones. Universal Illumina adapter and quality trimming of the sequencing reads was achieved using *Cutadapt* [44]. The trimmed reads were then aligned to the human reference genome (build GRCh37) using *bwa* [45] and then visualized using Integrative Genomics Viewer (IGV, Broad Institute). In parallel, to quantify the exact number of genetic variants for each CRISPR clone, we developed an R script that quantifies the percentage of each detected variant.

### Statistical analysis

Statistical analyses were performed using GraphPad Prism 5.04 (GraphPad Software, Inc., La Jolla, USA). Unpaired two-tailed parametric *t*-test or non parametric Mann Whitney test were carried out to compare two different conditions, as specified in the Figure Legends.

## Results

### ALDH1A2 and ALDH1A3 expression are enhanced in NB TICs

We have previously identified *ALDH1A2* and *ALDH1A3* genes as potential NB TIC markers as their expression was upregulated during NS selection of NB TICs derived from NB PDX tumors (181x and 9x baseline, respectively by Affymetrix microarray analysis) [17]. To confirm the enrichment in stem-like cells by serial NS passages of NB cell lines, the expression levels of various SC-associated markers were analyzed by RT-PCR and real-time PCR at different sphere passages in the SK-N-Be2c and NB1-C cell lines. NANOG, SOX2, and MYC mRNA expression levels were already increased at the second sphere passage (Additional file 1: Figure S1), confirming the rapid enrichment of NB TICs through serial NS culture.

**Fig. 1:**
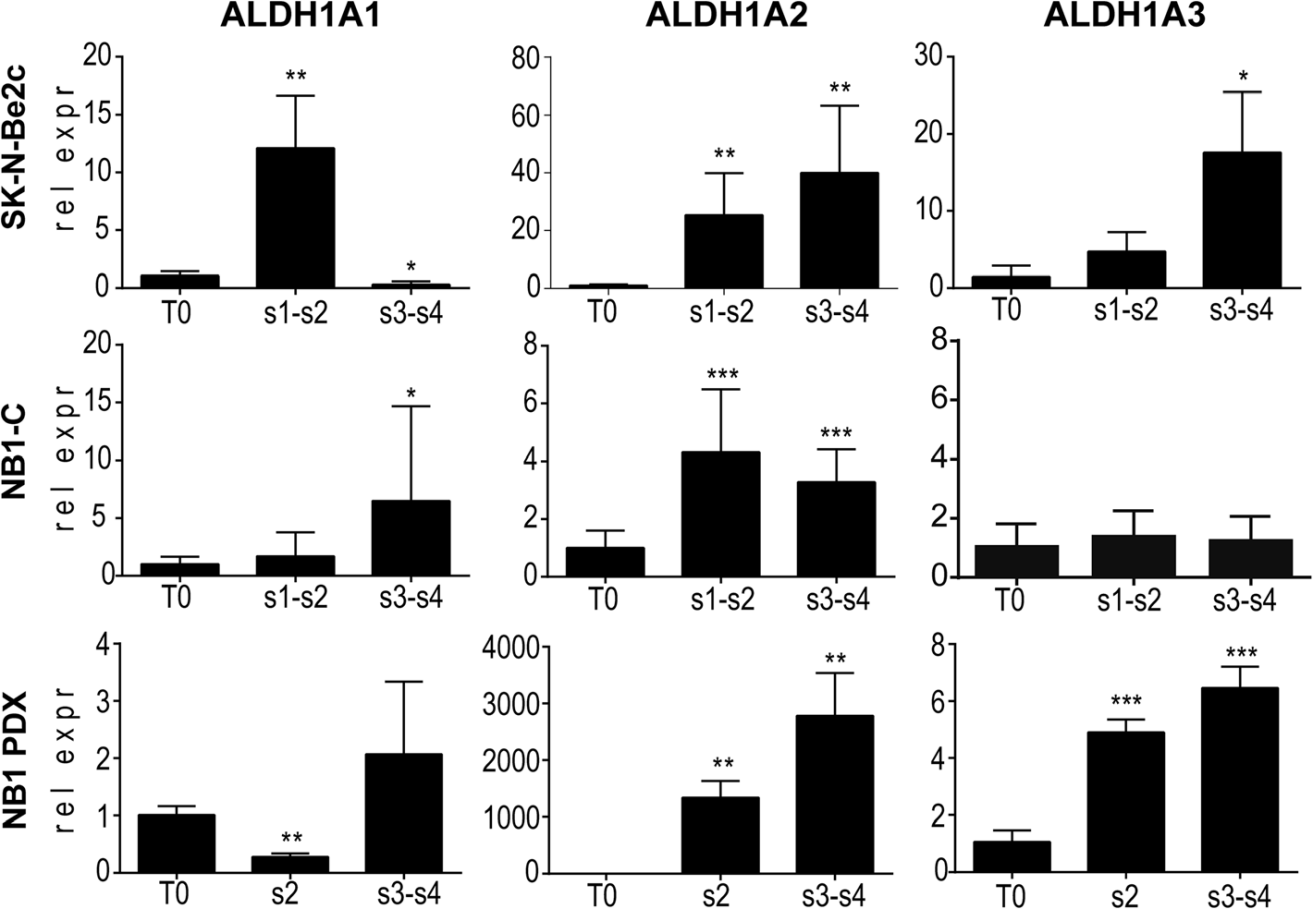
ALDH1A2 and A3 isoform expression are enhanced during NB self-renewal process. The mRNA expression levels of the three ALDH1 isoforms (ALDH1A1, ALDH1A2 and ALDH1A3) were analyzed by real-time PCR in parental cells (T0) and four sphere passages (s1 to s4) of SK-N-Be2c, NB1-C, and NB1 PDX-derived cells. Data are plotted as ALDH1 mRNA expression relative to the T0 parental cells in pooled s1-s2 and s3-s4 ± SD (unpaired *t*-test: * correspond to *p* < 0.05, ** *p* < 0.01, *** *p* < 0.0001)

To validate the prior microarray data and to provide a closer insight into ALDH1 isoenzyme expression status along the NB TIC selection, we analyzed the mRNA expression levels of the three ALDH1 isoforms in four successive NS passages and in parental cells (T0) growing in adherent conditions (Fig. 1). ALDH1A1 mRNA expression levels during TIC selection varied depending on the cell type and NS passages analyzed. In contrast, ALDH1A2 mRNA expression was strongly increased, in early steps of the TIC selection process (s1-s2) and remained elevated in later NS passages (s3-s4) in the I-type SK-N-Be2c cell line, as well as in the NB1 PDX tumor and the related NB1-C cell line (Fig. 1). Moreover, ALDH1A3 mRNA expression was also highly upregulated in the NB1 PDX tumor and in the SK-N-Be2c cell line during TIC selection, while it remained stable in the NB1-C cell line (Fig. 1). Altogether, these results confirm the enrichment of ALDH1A2 and ALDH1A3 mRNA expression observed in the NS microarray profiling derived from the NB PDX tumors [17].

**Fig. 2:**
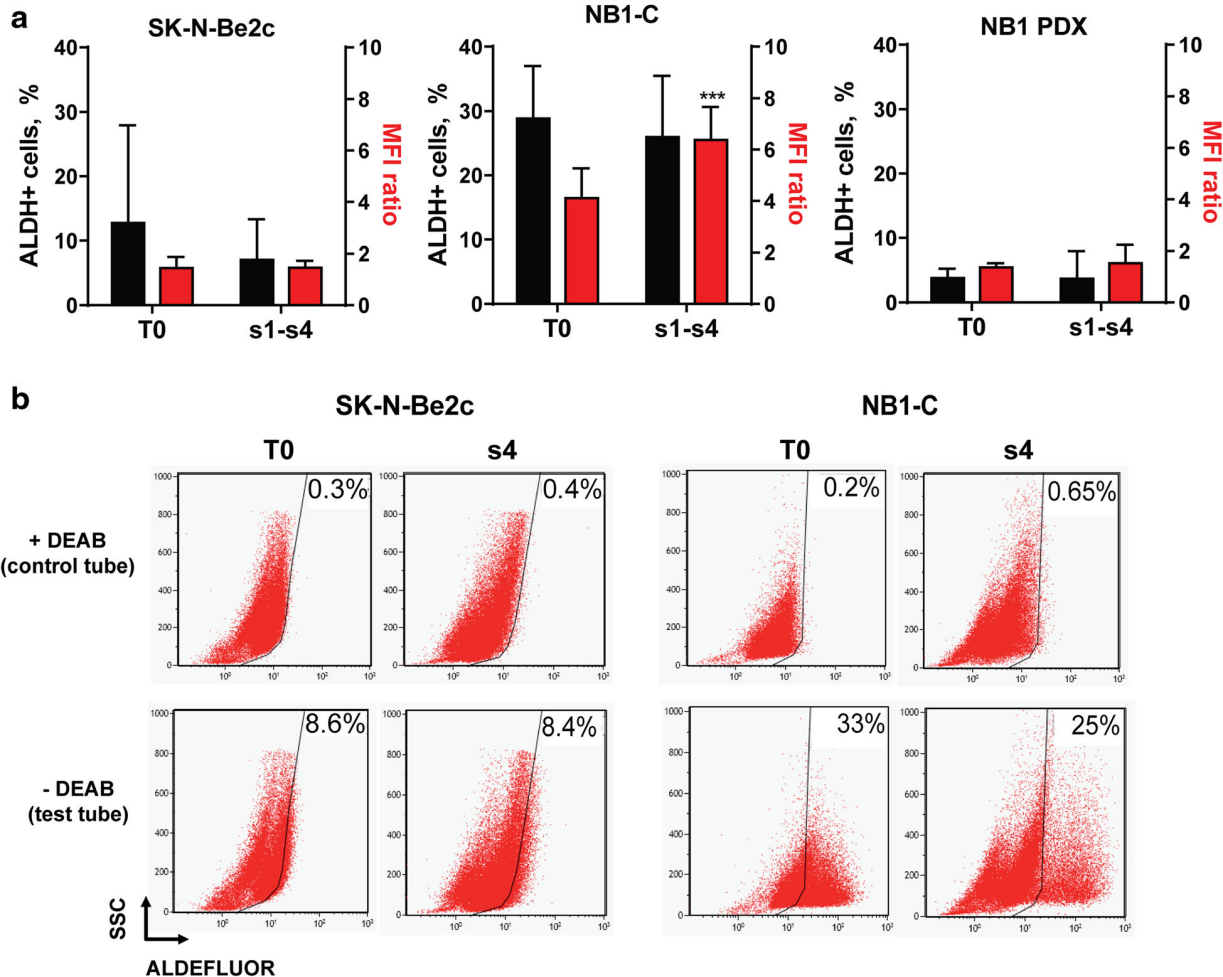
ALDH activity remains stable during NB TICs selection. ALDH activity was measured using the ALDEFLUOR kit in parental cells (T0) and successive sphere passages (s1 to s4) in SK-N-Be2c, NB1-C, and NB1 PDX-derived cells. **a** The percentage of ALDH^+^ cells (*black bars*) and the mean fluorescence intensity (MFI) ratio (MFI of the test tube/control tube, red bars) are plotted as mean ± SD of more than 3 experiments according to cell availability at each sphere passage (unpaired t-tests: ***p < 0.0005). **b** Representative dot plots showing the ALDH activity in SK-N-Be2c and NB1-C parental cells (T0) and in sphere passages 4 (s4) in presence (control tube) or absence (test tube) of DEAB. The percentage of ALDH^+^ cells are indicated in the dot plots

ALDH activity was also measured during successive NS passages from T0 to NS passage 4 (s4) in the SK-N-Be2c and NB1-C cell lines, and in cells derived from the NB1 PDX. The percentage of ALDH^+^ cells remained stable during TIC selection in all cells analyzed; similarly, the relative fluorescence intensity remained more or less constant during NS passages except for a slight increase in the NB1-C cell line (Fig. 2a and b). These results indicate a lack of correlation between ALDH activity (as measured using the ALDEFLUOR kit) and ALDH1 isoform expression in NB TICs (see Discussion).

### NB cell lines and PDX tumors display cell-specific ALDH1 isoenzyme expression profiles and elevated ALDH activity

**Fig. 3:**
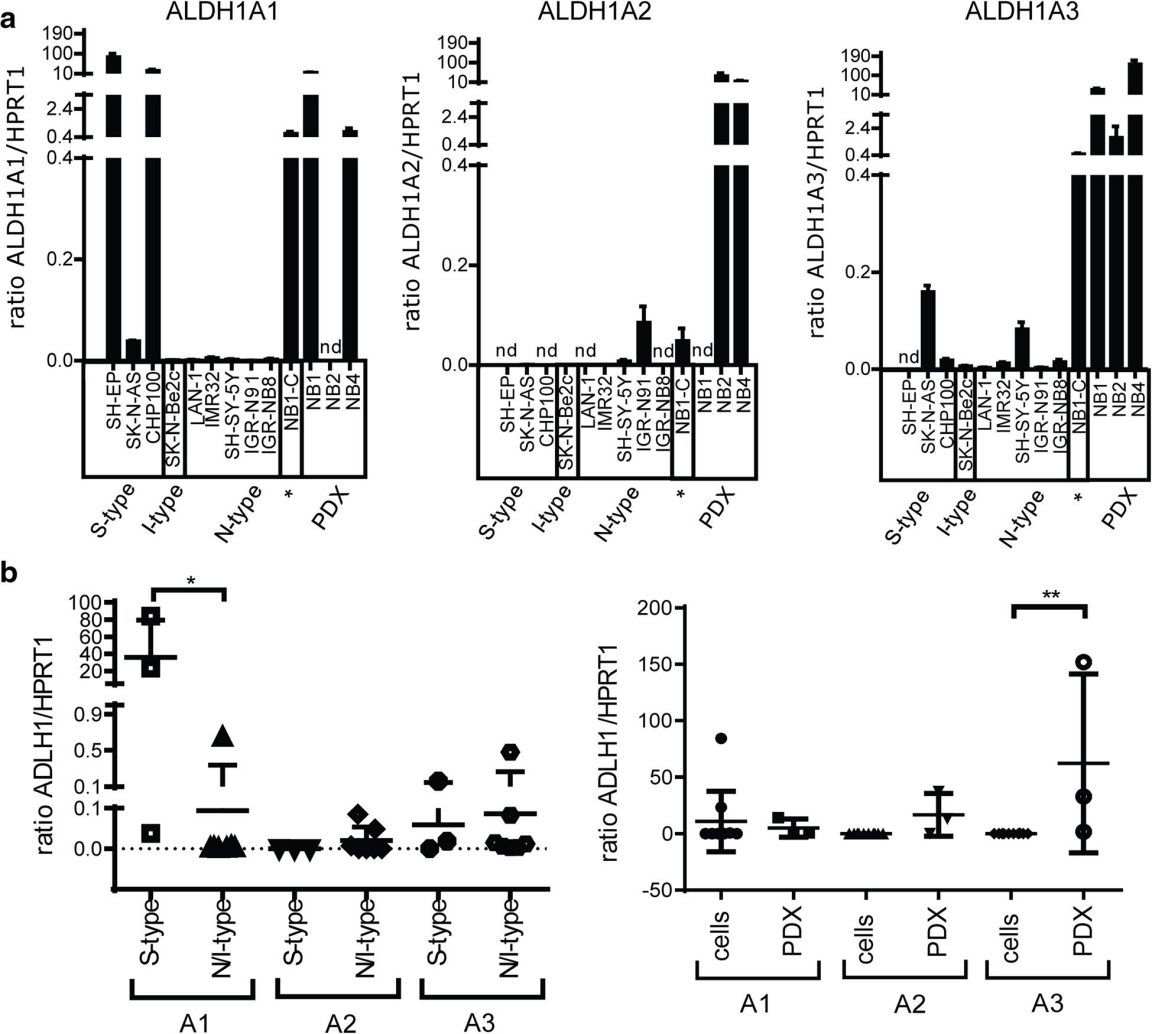
NB cell lines and PDX tumors display various ALDH isoenzyme expression patterns. **a** Basal endogenous mRNA expression levels of each ALDH1 isoform were measured in 10 NB cell lines (* = serum-free medium) and 3 NB PDX tumors by real-time PCR. Mean ratio of ALDH1 isoform/HPRT1 ***±*** SD are plotted in the bar graphs. Experiments were performed in tri- or quadruplicates (nd: not detected). **b** Comparison of ALDH1A1/A2/A3 mRNA expression levels between S-type versus N/I-type cell lines (*left panel*) and NB cell lines versus PDX tumors (*right panel*). Individual values and mean values ***±*** SD of ALDH1 isoform/HPRT1 ratio are plotted in the dot plots (Mann Whithney test *p = 0.033, **p = 0.007)

Next, the expression profile of each ALDH1 isoform was evaluated by real-time PCR in a large panel of NB cell lines and NB PDX tumors. Most NB cell lines analyzed expressed ALDH1A1 and/or ALDH1A3, but rarely ALDH1A2 (Fig. 3a). The less aggressive S-type cell lines expressed significantly higher levels of ALDH1A1 mRNA relative to N/I-type NB cells (Fig. 3a and b). Interestingly, the NB1-C cell line and the related NB1-PDX tumor displayed a similar expression pattern of ALDH1 isoenzymes, with elevated expression levels of ALDH1A1 and A3. Furthermore, the expression level of ALDH1A3 was significantly enhanced in the three PDX tumors as compared to NB cell lines, suggesting a role of this isoenzyme in in vivo grown tumors (Fig. 3a and b).

**Fig. 4:**
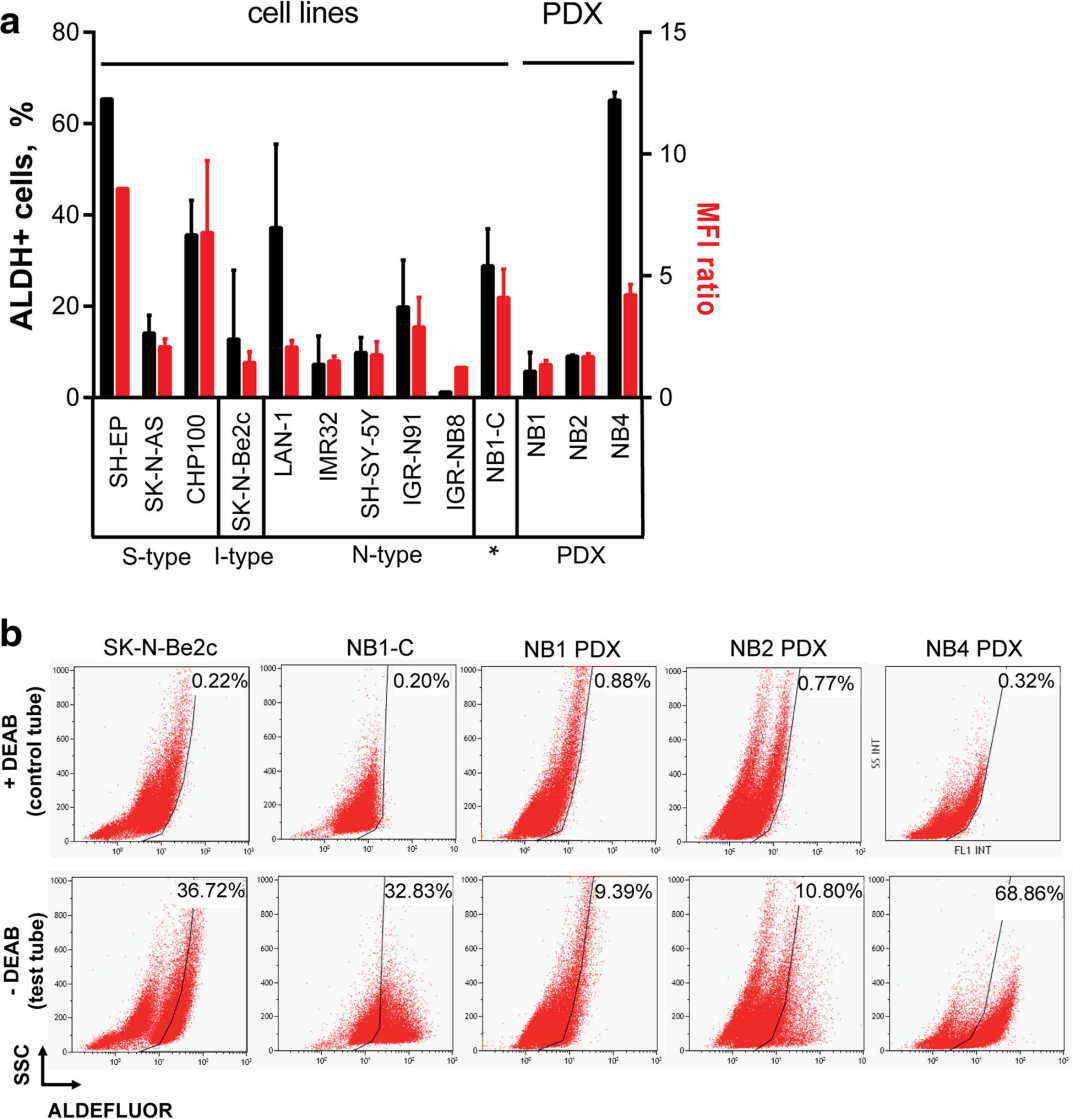
Heterogeneous ALDH activity is detected in NB cell lines and PDX tumors. **a** The percentage of ALDH^+^ cells (black bars) and the MFI ratio (MFI of the test tube/control tube, red bars), measured using the ALDEFLUOR kit, are given as mean ± SD of 1 to 8 experiments for the NB cell lines and 3 to 4 experiments for the PDX-dissociated tumor cells. **b** Representative dot plots showing the ALDH activity in SK-N-Be2c, NB1-C cell lines, and cells dissociated from the 3 PDX tumors in presence (control tube) or absence of DEAB (test tube). The percentages of ALDH^+^ cells are indicated

Further analysis of ALDH activity revealed elevated, yet heterogeneous, percentages of ALDH^+^ cells in NB cell lines and PDX tumors, ranging from 1.2 to 69 % (Fig. 4a and b).

### High ALDH1A3 expression in NB tumors correlates with poor outcome and high-risk prognostic markers

**Fig. 5:**
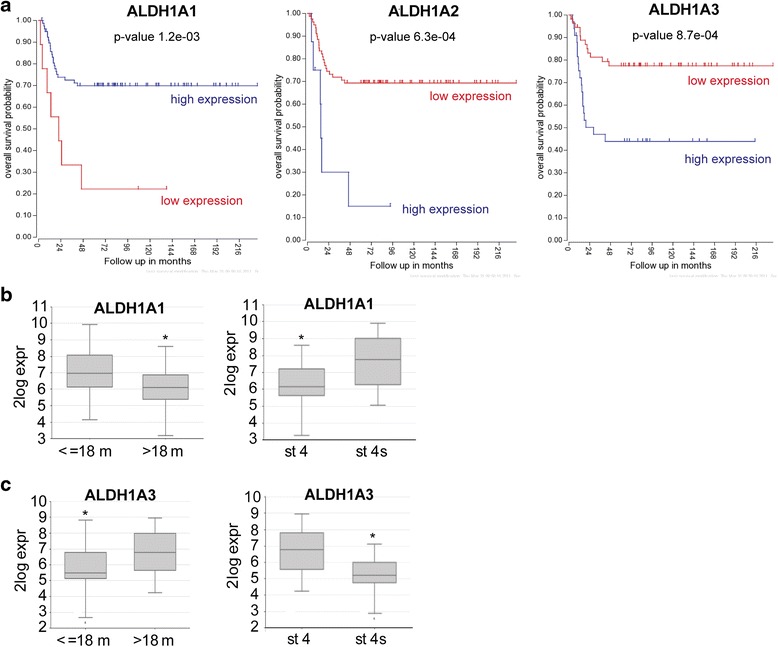
ALDH1A3 expression is associated with unfavorable prognostic markers and reduced survival, while ALDH1A1 expression correlates with less aggressive NB. **a**-**c** Graphs were generated from the Versteeg database (*n* = 88 NB patients) using the R2: Genomics Analysis and Visualization Platform (http://r2.amc.nl). **a** Kaplan Meier overall survival curves. **b**-**c** ALDH1A1 (**b**) and ALDH1A3 (**c**) mRNA expression levels in NB tumors according to age ≤ or > 18 month at diagnosis (*left panels*) or with stage 4 versus stage 4 s (*right panels*)

To identify the link between ALDH1 expression and NB aggressiveness, the ALDH1A1/A2/A3 expression patterns were analyzed in NB tumors using the R2: Genomics Analysis and Visualization Platform (http://r2.amc.nl). Analysis of the published dataset of Versteeg and colleagues [5] revealed that high ALDH1A1 expression is significantly associated with good prognosis, while elevated expression of ALDH1A3 is strongly correlated with poor survival (Fig. 5a). The expression level of ALDH1A2 is globally reduced in NB tumors compared to ALDH1A1 and A3 isoforms, but the rare tumors with high ALDH1A2 expression level displayed a very poor survival rate (Fig. 5a). Moreover, low expression levels of ALDH1A1 (Fig. 5b) or high expression levels of ALDH1A3 (Fig. 5c) are associated with unfavorable prognostic factors in NB (i.e., age at diagnosis >18 months, and stage 4 disease).

### Inhibition of ALDH activity affects NB aggressive properties

**Fig. 6:**
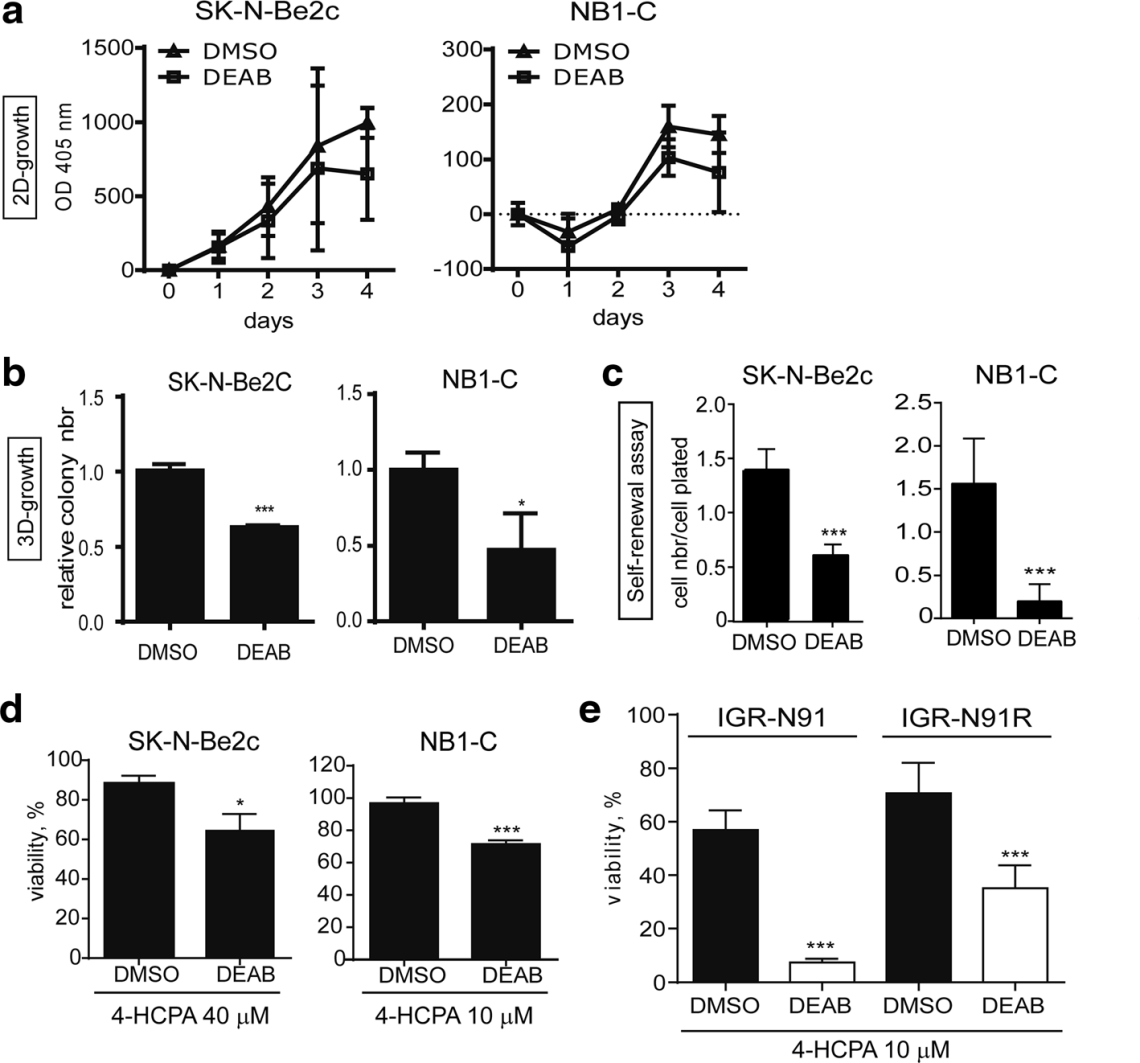
DEAB-mediated ALDH inhibition affects NB aggressive properties and sensitizes NB cells to 4-hydroxycyclophosphamide. Analyses of the impact of ALDH activity inhibition by DEAB treatment on NB cell proliferation (2D-growth, **a**), clonogenicity (3D-growth, **b**), TICs self renewal (**c**), and sensitivity to 4-HCPA (**a**-**e**). SK-N-Be2c and NB1-C cell lines were pre-treated with 100 or 50 μM of DEAB, respectively, for three days before starting functional assays performed in presence of DEAB or DMSO as control. **a** Mean OD at 405 nm ± SD of 4 (SK-N-Be2c) or 2 (NB1-C) experiments performed in quadruplicates. **b** Mean of relative colony numbers ± SD of 3 experiments performed in duplicates (unpaired *t*-test *p < 0.05, ***p ≤ 0.0005). **c** Mean ratio of cell number/cell plated ± SD of 2 experiments performed in triplicates (unpaired *t*-test ***p ≤ 0.0001). **d** Cell viability of SK-N-Be2c and NB1-C cells treated for 48 h with indicated doses of 4-HCPA in presence or absence of DEAB (100 μM and 50 μM, respectively). Mean values ± SD of 3 experiments performed in quadruplicates (unpaired *t*-test *p < 0.02, ***p ≤ 0.0001). **e** Cell viability of IGR-N91 and IGR-N91R cells treated for 48 h with indicated doses of 4-HCPA in presence or absence of DEAB (100 μM). Mean values ± SD of 3 experiments performed in quadruplicates (unpaired *t*-test ***p ≤ 0.0005)

We next investigated whether the inhibition of ALDH activity in NB cell lines affects the NB cell properties associated with aggressiveness, such as proliferation, anchorage-independent growth, and TIC self-renewal. First, we confirmed that subtoxic doses of DEAB, a well-known specific inhibitor of ALDH activity [21], fully inhibit ALDH activity, which can be recovered by DEAB removal (Additional file 1: Figure S2). Treatment with DEAB had no impact on the 2D-proliferation capacities of SK-N-Be2c and NB1-C cell lines (Fig. 6a). However, ALDH activity inhibition strongly affected NB cell clonogenic properties (Fig. 6b), and negatively impacted on TIC self-renewal capacities of SK-N-Be2c and NB1-C cell lines by 43 and 88 %, respectively (Fig. 6c).

As ALDH activity was shown to mediate resistance to 4-hydroxycyclophosphamide (4-HCPA), NB cell sensitivity to this chemotherapeutic agent was also measured in presence or absence of DEAB. We observed that ALDH inhibition partly sensitized SK-N-Be2c and NB1-C cell lines to 4-HPCA (Fig. 6d). In addition, we analyzed two cell lines, the drug-sensitive IGR-N91, and the multidrug resistant IGR-N91R, previously established in our lab [35]. Interestingly, ALDH enzymatic inhibition was able to almost completely sensitize the IGR-N91 cells to 4-HCPA and had a strong sensitizing impact on the multidrug resistant IGR-N91R cells (Fig. 6e). Altogether these results demonstrate that endogenous ALDH activity plays a role in NB cell aggressive properties and mediates NB cell resistance to the chemotherapeutic drug 4-HCPA.

### ALDH1A3 knock out affects NB clonogenic properties

**Fig. 7:**
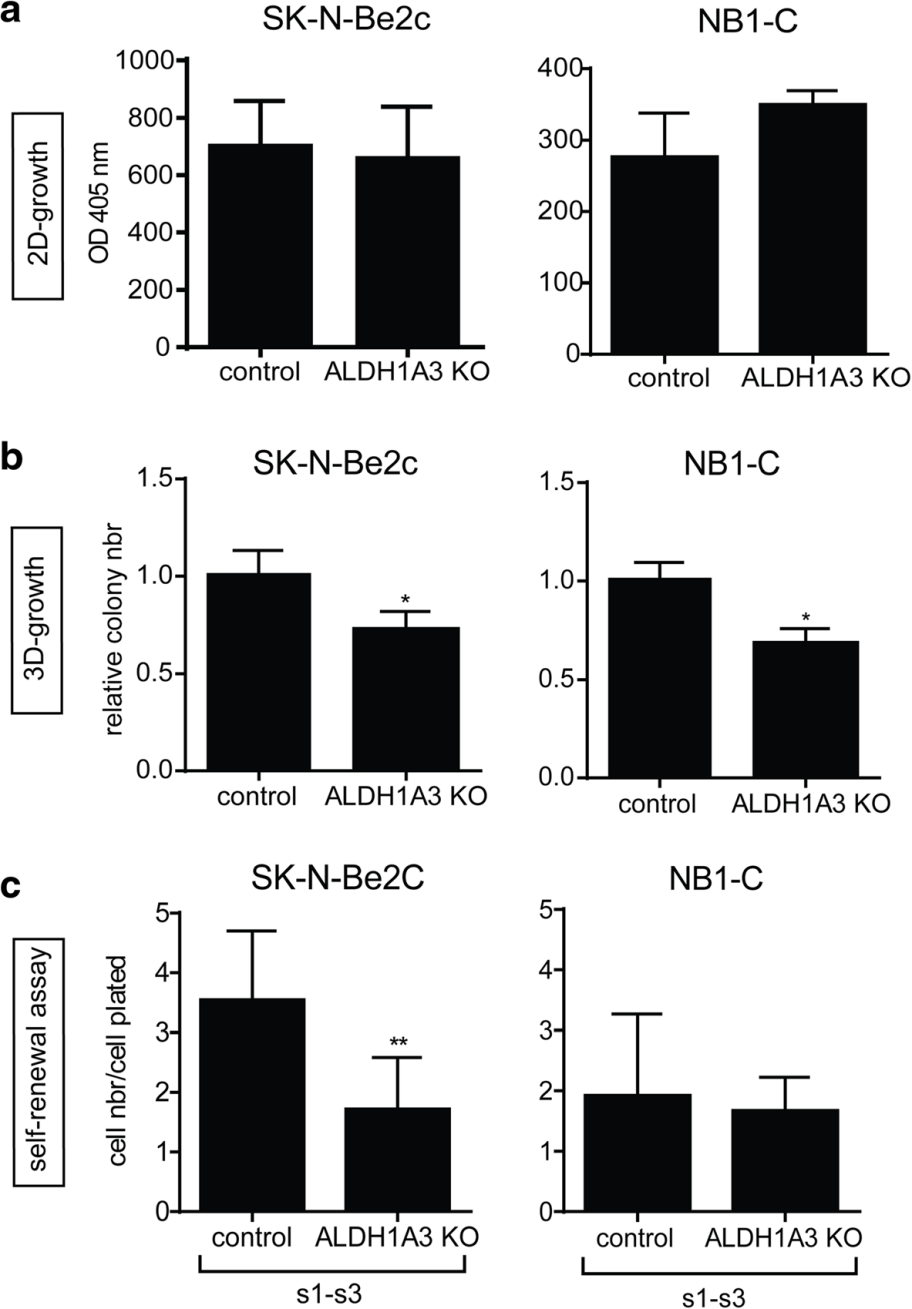
Specific ALDH1A3 KO impairs NB cell clonogenic properties. **a**-**c** Impact of ALDH1A3 specific KO was analyzed on the proliferation (**a**), clonogenic (**b**), and TIC self-renewal (**c**) capacities. **a** Mean OD at 405 nm ± SD of 3 experiments performed in quadruplicates. **b** Mean of relative colony numbers ± SD of 3 experiments performed in duplicates (unpaired *t*-test *p < 0.05). **c** Mean ratio of cell number/cell plated ± SD of 3 experiments performed in duplicates in pooled s1 to s3 passages (s1-s3) (unpaired *t*-test **p ≤ 0.005)

Although ALDH activity measured by the ALDEFLUOR assay was initially mainly attributed to the ALDH1A1 isoform, other ALDH isoenzymes, such as ALDH1A2, ALDH1A3, ALDH2, ALDH3A1, and ALDH9A1, could be involved in the measured ALDH activity [21, 25, 28, 29]. As the ALDH1A3 isoform is associated with poor survival in NB and is the most widely expressed ALDH1 isoform in our panel of NB cell lines and PDX tumors (Figs. 5a and 3a, respectively), we asked whether ALDH1A3 activity plays a functional role in NB aggressive phenotype. To answer this question, ALDH1A3 knock-out (KO) SK-N-Be2c and NB1-C cell lines were generated by CRISPR/Cas9 gene editing (Additional file 1: Figure S3). Similarly, as for the observations after DEAB-mediated ALDH inhibition, ALDH1A3 KO did not affect the 2D-cell proliferation properties, but decreased the 3D-anchorage-independent growth of both NB cell lines (Fig. 7a and b). While, ALDH1A3 KO only impaired the TIC self-renewal properties of the SK-N-Be2c cell line, but had no effect on NB1-C cells (Fig. 7c).

## Discussion

In this study, we first confirmed the enrichment of ALDH1A2 and ALDH1A3 expression during NB TICs selection in one PDX tumor (NB1), as well as in two distinct cell lines, the NB1-C cells derived from the NB1 PDX tumor, and/or the I-type SK-N-Be2c cell line. The enhancement of ALDH1A2 and ALDH1A3 mRNA expression levels during TIC selection of NB1-PDX derived cells was in accordance with the fold increase (181x and 9x, respectively) as observed in our previous microarray analysis [17]. Interestingly, the implication of ALDH1A2 in the regulation of CSC properties in NB has recently been reported [33]. Moreover, high ALDH1A2 expression in NB correlates with poor survival, suggesting a role for this ALDH1 isoenzyme in NB tumor aggressiveness.

ALDH activity has been demonstrated to select CSCs in leukemia and breast, lung, liver, prostate, brain, and colon cancer [23–25]. However, despite significant overexpression of ALDH1A2 and ALDH1A3 during TIC selection, no increase in the percentage of ALDH^+^ cells could be observed during NS-passages by ALDEFLUOR assay measurements. This suggests that ALDH enzymatic activity may not be a valuable functional marker of TICs in NB. Similar findings were previously described in melanoma [46].

Our analysis of ALDH1 isoform expression profiles in NB cell lines and PDX tumors revealed differential expression patterns which may rely on the strong heterogeneity of NB tumors and cell lines. The higher expression levels of ALDH1A1 in the less aggressive S-type cell lines is in accordance with the finding that elevated ALDH1A1 expression in NB tumors correlates with a better survival rate and favorable prognostic factors. In other neoplasms, ALDH1A1 has been shown to correlate either with favorable or poor prognosis depending on the tumor setting or on tumor sample sets [23]. Moreover, we observed that ALDH1A3 is strongly expressed in PDX tumors, and that higher ALDH1A3 expression correlates with poor survival and high-risk prognostic markers. These observations, as well as the ALDH1A3 enrichment in NB TICs, suggest that ALDH1A3 isoenzyme could be linked to NB progression and aggressiveness. This correlates with other studies showing that high ALDH1A3 expression is associated with more aggressive forms of breast, glioblastoma, glioma, and pancreatic cancer [28, 47–49].

NB PDX tumors and cell lines also displayed a strong and heterogeneous ALDH enzymatic activity. However, no correlation between the expression levels of a specific ALDH1 isoform and ALDH activity could be identified in these samples, as well as during NB TIC selection. Although the ALDH enzymatic activity measured by the ALDEFLUOR kit was initially mainly attributed to ALDH1A1, other ALDH isoenzymes were also involved [21, 25, 28, 29]. Further investigations are needed to determine if the ALDH activity detected in NB cells can be associated with a specific ALDH isoenzyme. Yet, we observed that *ALDH1A3* gene disruption had no major impact on the ALDH enzymatic activity in SK-N-Be2c and NB1-C clones (Additional file 1: Figure S4). These data suggest that ALDH1A3 isoenzyme play a negligible role in the conversion of the ALDH substrate, BODIPY-aminoacetaldehyde, to fluorescent BODIPY-aminoacetate reaction products in NB cells, in contrast to breast cancer and non-small cell lung carcinoma [28, 29].

Drug resistance is a hallmark of CSCs or TICs and is considered as a major contributing factor of relapse. Various mechanisms of chemoresistance have been identified in CSCs, including ALDH activity [50]. Indeed, ALDH activity has been associated for a long time with normal SC and CSC resistance to oxazaphosphorines such as cyclophosphamide [23, 25, 51–53], a drug commonly used during NB patient therapy. A cyclophosphamide-resistant phenotype in relation with high ALDH activity has not yet been described in NB. In this study, we demonstrate a significant sensitization of NB cell lines to 4-HCPA, the active metabolite of cyclophosphamide, upon ALDH activity inhibition with DEAB, which represents an original finding. Importantly, the multidrug resistant IGR-N91R cells could be efficiently resensitized to 4-HCPA using DEAB. As DEAB was shown to most potently inhibit ALDH1A1, followed by ALDH2, ALDH1A2, ALDH1B1, ALDH1A3 and ALDH5A1 [54], further studies will be required to determine the specific involvement of individual ALDH isoenzyme in ALDH-mediated resistance to 4-HCPA in NB.

Interestingly, we also demonstrate in this present study that treatments of NB cells with DEAB induced a significant decrease in their anchorage-independent growth and TIC self-renewal properties. *ALDH1A3* gene disruption also impaired the clonogenic properties of both cell lines analyzed. However, *ALDH1A3* KO only affected the TIC self-renewal capacities of the SK-N-Be2c cell line, but not that of NB1-C cells. This observation correlates with the lack of enrichment of the ALDH1A3 isoenzyme in the NB1-C cell line during self-renewal assays, in contrast to ALDH1A3 enrichment in the SK-N-Be2c cells and the NB1 PDX-derived cells (Fig. 1). This suggests that the ALDH1A3 isoenzyme may not play a major role in TIC self-renewal in the NB1-C cell line.

The limitations of the present work are partly due to the difficulty to precisely assess both global ALDH activity and particular isoform inhibition. This is an issue shared by all prior reports on ALDH function. In fact, the ALDEFLUOR kit has been thought to faithfully measure ALDH1A1 isoform activity, a presumption which has subsequently been shown not be entirely correct (see above). Moreover, DEAB selectively inhibits specific ALDH isoenzymes, and this at various levels of efficiency. In this study, we addressed these limitations by performing genetic knock-outs that (from our previous work on TICs) appeared to influence the biology of NB, and investigated their impact. Similarly, as for other metabolic pathways involved in oncogenesis, the functional redundancy of multiple enzymatic isoforms limits the investigation of the mechanistic underpinnings behind the effects we and other observed. Nonetheless, we feel that, while our findings are mostly focused on in vitro assays for oncogenic potential, this study opens new avenues for in vivo investigations. In particular, the ALDH-associated re-sensitization of NB to frequently used chemotherapeutic agents (i.e., cyclophosphamide) will need to be further detailed as it constitutes a potential translational avenue. In addition, the data linking ALDH variation with clinical outcomes in NB patients correlate with our prior work and the current results. We feel they constitute an encouraging step towards further work addressing the role of ALDH isoforms in murine models of NB, in particular PDX.

## Conclusions

Our results highlight the impact of ALDH enzymatic activity on the aggressive properties of NB in addition to its resistance to 4-HCPA. Further work is needed to identify the specific ALDH isoenzyme(s) involved as they may be considered for future therapeutic strategies for high-risk NB.

## Additional file

Additional file 1: Figure S1. Stem cell markers are enriched during neurosphere culture. **Figure S2.** DEAB treatment is efficient to transitory inhibit ALDH activity. **Figure S3.** Illustration of the insertions/deletions in the different ALDH1A3 KO clones. **Figure S4.** ALDH1A3 KO has no impact on the percentage of ALDH+ cells. (ZIP 3370 kb)

## Abbreviations

4-HCPA: 4-hydroxycyclophosphamide
ALDH: Aldehyde dehydrogenases
CSCs: Cancer stem cells
DEAB: Diethylaminobenzaldehyde
DMSO: Dimethyl sulfoxide
MFI: Mean fluorescence intensity
NB: Neuroblastoma
NBM: Neural basic medium
NCSC: Neural crest stem cell
NS: Neurospheres
PDX: Patient-derived xenograft
TICs: Tumor-initiating cells
