# Supplementary material for: Aldehyde dehydrogenase activity plays a Key role in the aggressive phenotype of neuroblastoma
Source: BMC Cancer. 2016 Oct 10;16:781. doi: 10.1186/s12885-016-2820-1 (PMC5057398; doi:10.1186/s12885-016-2820-1)
Supplement: Additional file 1: Figure S1. — Stem cell markers are enriched during neurosphere culture. Figure S2. DEAB treatment is efficient to transitory inhibit ALDH activity. Figure S3. Illustration of the insertions/deletions in the different ALDH1A3 KO clones. Figure S4. ALDH1A3 KO has no impact on the percentage of ALDH+ cells. (ZIP 3370 kb) [file 12885_2016_2820_MOESM1_ESM.zip › Additional Files Figure legends.docx]

**Additional Files**

**Fig S1.** **Stem cell markers are enriched during neurosphere culture.** (A) The expression levels of the stem cell markers Nanog, Sox2 and Myc were analyzed by RT-PCR in total RNA obtained from SK-N-Be2c and NB1-C parental cells (T0) and the sphere passage s2. The *HPRT1* gene was used as control. (B) MYC mRNA expression was analyzed by real-time PCR in the sphere passages s2, and s5 relative to parental cells (T0). Mean relative expression ± SD of two experiments performed in duplicates were plotted in the bare graph (unpaired t-test, *p<0.05).

**Fig S2. DEAB treatment is efficient to transitory inhibit ALDH activity.** ALDH activity was analyzed in untreated SK-N-Be2c cells, after prolonged treatment with 100 μM DEAB for 5 days, or after 3 days in presence of DEAB followed by 2 days in absence of DEAB. Representative dot plots are shown.

**Fig S3. Illustration of the insertions/deletions in the different ALDH1A3 KO clones**. DNA sequences of WT *ALDH1A3* gene and mutated alleles of 2 clones of SK-N-Be2c cells and 2 clones NB1-C cells identified by MiSeq Illumina sequencing. Insertions/deletions are indicated in red. The sgALDH1A3.1 and sgALDH1A3.2 are highlighted in light grey and in dark grey, respectively, and the PAM sequence is labeled in black bold. The number of reads and percentage of each allele/total number of reads are indicated, as well as the total coverage. Note that three alleles were found in the SK-N-Be2c clone 1.9, indicating a triploidy of this genomic region, which was confirmed by the ratio of 2 different indels found in clone 1.18. The alleles 1 and 2 have the same indel. In addition, only one indel could be identified in each NB1-C clone, suggesting LOH in this genomic region. All indels lead to premature stop codons in the N-term end of the ALDH1A3 protein as indicated.

**Fig S4. ALDH1A3 KO has no impact on the percentage of ALDH^+^ cells.** ALDH activity was measured using the ALDEFLUOR kit in control and ALDH1A3 KO SK-N-Be2c and NB1-C clones. (A) The percentage of ALDH^+^ cells are plotted as mean ± SD of 2 experiments.
