# Supplementary figures and images for: Aldehyde dehydrogenase activity plays a Key role in the aggressive phenotype of neuroblastoma
Source: BMC Cancer. 2016 Oct 10;16:781. doi: 10.1186/s12885-016-2820-1 (PMC5057398; doi:10.1186/s12885-016-2820-1)

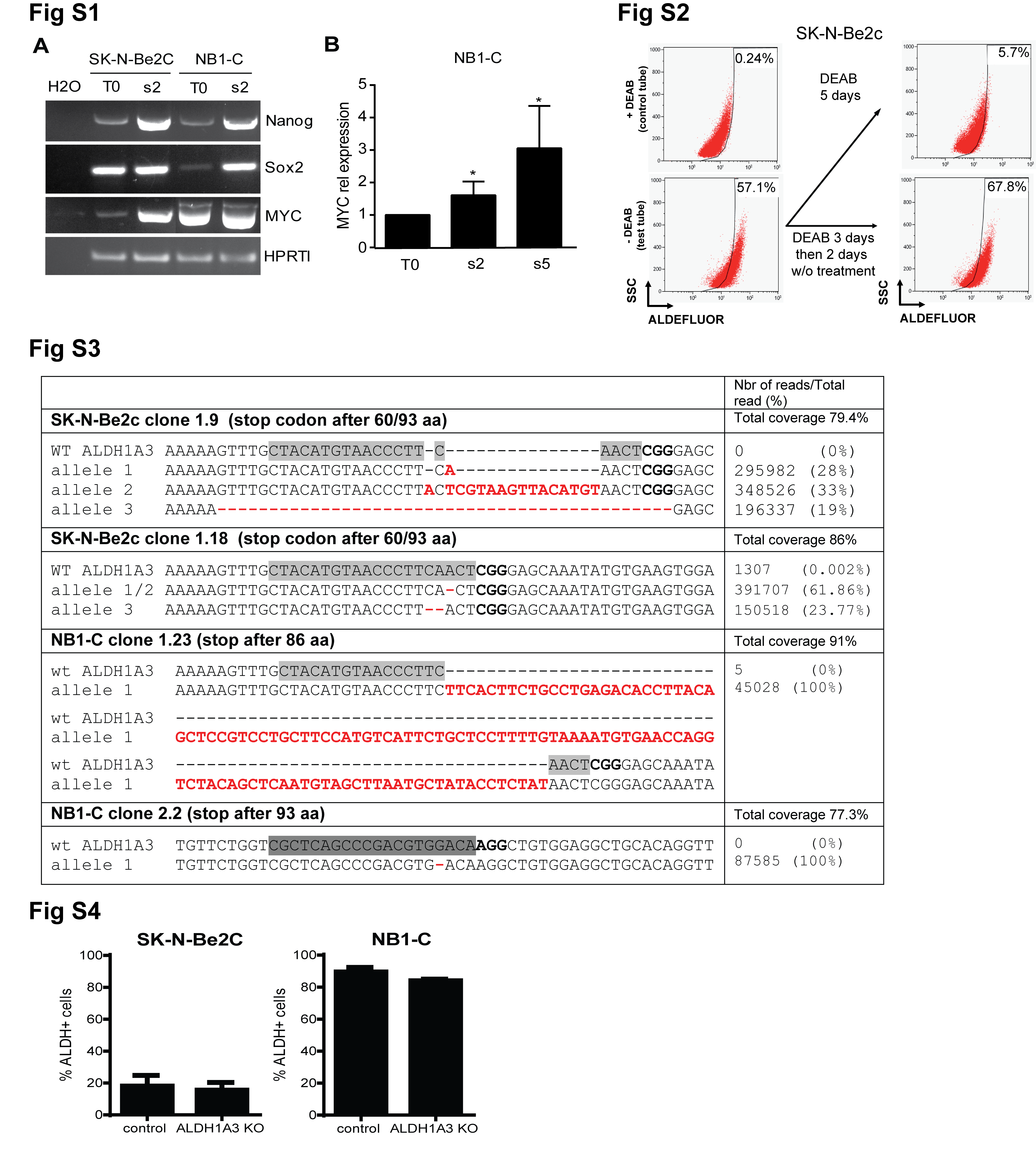

Supplement: Additional file 1: Figure S1. — Stem cell markers are enriched during neurosphere culture. Figure S2. DEAB treatment is efficient to transitory inhibit ALDH activity. Figure S3. Illustration of the insertions/deletions in the different ALDH1A3 KO clones. Figure S4. ALDH1A3 KO has no impact on the percentage of ALDH+ cells. (ZIP 3370 kb) [file 12885_2016_2820_MOESM1_ESM.zip › Additional Figures.tif]
